# Kindlins as modulators of breast cancer progression

**Published:** 2021

**Authors:** Edward F. Plow, Elzbieta Pluskota, Katarzyna Bialkowska

**Affiliations:** Department of Cardiovascular and Metabolic Sciences, Lerner Research Institute, Cleveland Clinic, 9500 Euclid Ave, Cleveland, OH, USA 44139

**Keywords:** Kindlins, FERMT, FERM domains, Integrins, Breast cancer, Talin

## Abstract

Kindlin-1 (K1, FERMT1), Kindlin-2 (K2, FERMT2), and Kindlin-3 (K3, FERMT3) are the three members of the kindlin family of adapter proteins found in mammals. One or more kindlins are found in most cell types, K1 primarily in epithelial cells, K3 in primarily hematopoietic cells and also endothelial cells, and K2 is very broadly distributed. The kindlins consist primarily of a 4.1-erzin-radixin-moiesin (FERM) domain, which is transected by a lipid-binding plextrin-homology (PH) domain. Deficiencies of each kindlin in mice and/ or humans have profound pathogenic consequences. The most well-established function of kindlins depends on their ability to participate in the activat integrin adhesion receptors. This function depends on the binding of each kindlin to the beta subunit of integrins where it cooperates with talin to enhance avidity of interactions with cognate extracellular matrix ligands. Deficiencies of many different integrins are lethal, are critical for normal development of mammary tissue, and excessive expression and/or activation of certain integrins are associated with progression and metastasis of breast cancer. However, via its interaction with many other intracellular proteins, kindlins can influence numerous cellular responses. Changes in expression of each of the three kindlins have been reported in association with breast cancer, with several studies indicating that kindlins are among the most upregulated genes in breast cancer. The association of abnormal functions of K2 with breast cancer is particularly extensive with many reports indicating that it is a major driver of breast cancer via its promotion of cancer cell proliferation, survival, adhesion, migration, invasion, the epithelial-to-mesenchymal transition and its influence on macrophage recruitment and phenotype. These associations suggest that the kindlins and their functions represent an intriguing therapeutic target for exploration of breast cancer therapy.

## Introduction

### Kindlin structure

**Figure 1:**
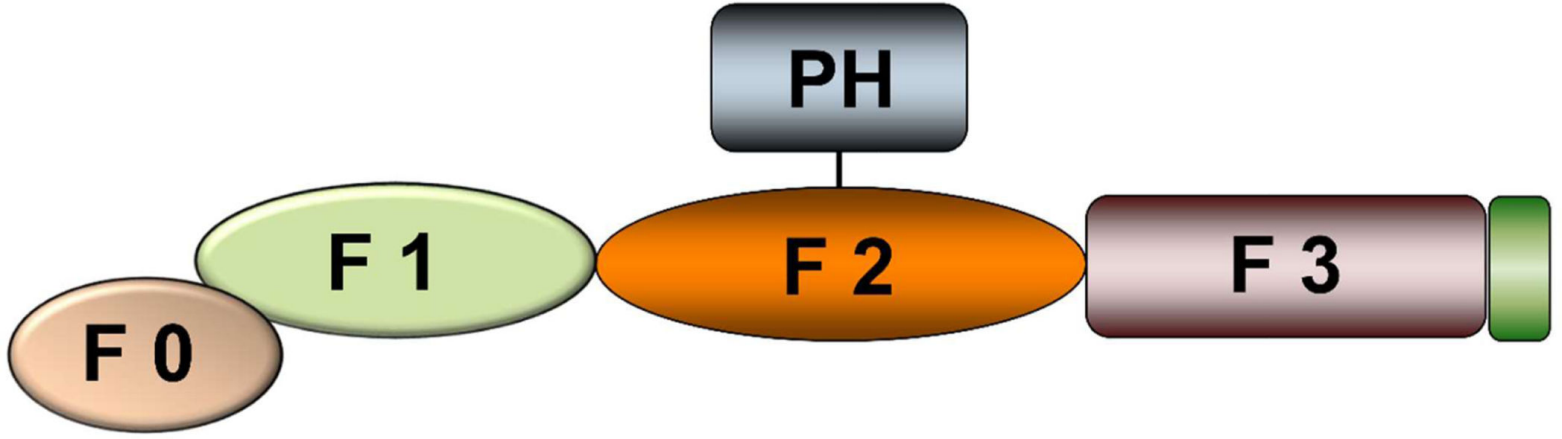
Diagram illustrating the organization of a typical Kindlin. It is composed of a FERM domain, consisting of the three F1, F2 and F3 subdomains. It is preceded by a F0 subdomain and a PH domain transects the F2 subdomain. The typical Kindlin has a molecular weight of ~75,000.

This review considers the roles of the three kindlin family members, kindlin-1 (K1), kindlin-2 (K2) and kindlin-3 (K3) in initiation, progression and metastasis of breast cancer. Kindlins are also referred to by their gene names, FERMT1, FERMT2, and FERMT3, so named for their content of a 4.1-ezrin-radixin-moesin (FERM) domain. FERM domains are common building blocks of proteins, and the FERM family contains more than 50 members [1]. In the three kindlins found in mammals, the FERM domain is their dominant structural feature. For convenience, the subdomains of kindlins are often displayed in a linear fashion with F1 near the N-terminus, F2 in the middle, and the F3 subdomain, which is a phospho-tyrosine-like binding (PTB-like) domain, at the C-terminus (Figure 1). In all three kindlins, the F1 subdomain is preceded by an N-terminal F0 subdomain, which adopts a ubiquitin-like fold [2]. Recently solved crystal structures of kindlins show that the subdomains fold independently into a four-lobed structure [3–5]. The three kindlins in mammals are each about 70 kDa and are ~50% identical at an amino acid level with regions of high conservation with interspersed variable regions that account for the common and individualized functions of the kindlins [6]. The three kindlins seem to have evolved via gene duplication with evolutionary pressure toward specialization [7]. In terms of sequence similarities, kindlins are most similar to the FERM domain in talin, a large actin binding cytoskeletal protein that is necessary for activation of integrin adhesion receptors [8]. These similarities were an impetus for some early studies of kindlin function. Initial insights into kindlin function also emerged from studies of *C. elegans,* where the kindlin paralogue (*unc112)* was necessary for formation of muscle attachments to membrane that also contains ILK and integrin beta subunit paralogs [9]. These are now established binding partners of human kindlins, and each has been linked to breast cancer.

### Integrins as key participants in breast cancer progression

Cell adhesion to extracellular matrix and conjugation with other cells is essential to initiation, development and survival of all multicellular organisms, and members of the integrin family of heterodimeric transmembrane receptors are key mediators of many such cell-matrix/cell-cell interactions. Integrins are non-covalent heterodimers composed of α and β subunits with each containing a single transmembrane spanning segment. The combination of 18α and 8β subunits leads to formation of 24 integrins that are broadly distributed on both circulating and resident cells, where they mediate interactions with a variety of ECM proteins [10–12]. Key to participation of integrins in such interactions is their versatility: collectively, they recognize a diverse array of ligands; they transmit bidirectional signals, outside-in and inside-out, across cell membranes to initiate both intracellular and extracellular responses; and they can alter their affinities/avidities for cognate ligands to insure rapid and productive interactions. Such mechanisms allow integrins to participate in numerous physiological responses.

In the mammary gland, cellular interactions with ECM are crucial for adhesion, migration, proliferation, differentiation and survival of mammary epithelial cells, all indispensable for ductal morphogenesis and functional integrity of the gland [13,14]. However, the functions of integrins can be subverted to drive oncogenic transformations in the mammary epithelium, and multiple studies implicate integrins in both early and malignant breast cancer (e.g.[15]). As selected examples, targeted disruption of β1-integrins in a mouse model of human breast cancer leads to a dormant phenotype and blocks tumor induction [16] β4 integrins promotes mammary tumorigenesis *in vivo* via upregulation of ErbB2 signaling.[17]. Furthermore, integrins promote metastatic progression of breast cancer tumors. For example, the β1 integrin is indispensable for metastasis of Her2+ cancer *in vivo.* [18]. Integrin αVβ3, which is not expressed on normal mammary epithelium but is highly expressed on invasive breast cancer cells, is essential for breast cancer metastasis to bone. This effect is due in part to its interaction with a bone-derived glycoprotein osteopontin and to enhanced invasive potential due to upregulation and activation of metalloproteinases and the urokinase/urokinase receptor axis [19–21]. The αVβ3 has also been shown to promote EMT in epithelial breast cancer cells via crosstalk with TGF-β receptor signaling pathway [22]. In addition, integrins modulate functions of mammary stem cells. For example, genetic ablation of β1 integrins within basal myoepithelial cells attenuates stem cell renewal [23]. In cancer, α6 and β3 integrins are overexpressed in tumor-initiating cells and promote their self-renewal [24–26]. Integrins can also confer human breast cancer resistance to radiotherapy [27] and immunotherapy [28]. Systemic administration of a function blocking antibody to the β1-integrin subunit or a peptide inhibiting the αVβ1 and αVβ3 integrins significantly reduced the growth and metastasis of invasive human breast cancer cells in a mouse xenograft model [29,30].

### Functions of kindlins

The three kindlins exhibit differences in their expression profiles: kindlin-1 is expressed mainly in cells of epithelial origin; kindlin-2 is very broadly expressed; and kindlin-3 is expressed primarily in hematopoietic cells [31]. However, there are now known to be several exceptions to these primary localizations [32]. Notably, several recent studies have showed that the kindlin family members can be expressed in several human cancer cells, including in breast cancer cells [33–35]. Furthermore, more than one kindlin can be expressed within the same cell. As notable examples, K2 and K3 have been detected in endothelial cells [32] although this observation has not been reproduced in another study [36] and K1 and K2 can be detected in epidermis and colocalize in keratinocytes [37].

The most extensively studied and well-established function of kindlins relates to their regulation of integrin activation. Kindlins and talin cooperate to achieve optimal integrin activation (e.g. [2,38]). Ablation of either gene, blunts ligand recognition by integrin, and knockout of both genes can fully suppress ligand engagement by integrins. Kindlin and talin bind to adjacent but non-overlapping sites on the cytoplasmic tails of integrins [39]. The mechanism by which kindlin activates integrin is not fully resolved; kindlins have been implicated in integrin clustering [40], avidity modulation; it may involve conformational changes within a single integrin [38,41,42], affinity modulation, it may depend on linking talin and kindlin together via a common binding partner [43], or it may involve exertion of force on the integrin via the capacity to stimulate actin rearrangement [44]. Also, there is some ambiguity as to the definition of the term “activation”. Some integrins have high intrinsic activity so they can mediate adhesion to substrates without stimulation of the cells, but adhesion, spreading and signaling occurs more rapidly with integrin activation, whereas other integrins, such as those present on circulating cells, display minimal capacity to engage ligands without stimulation of the cells leading to integrin activation. However, if the role of kindlins were restricted to its binding and activation of integrins, only part of their role in breast cancer would be considered. Kindlins are adapter proteins. Numerous direct binding partners have been identified, and the list is under continued expansion [2,33]. Furthermore, the reach of kindlins broadens through its capacity to act as an assembly hub within cells. Many of the binding partners of kindlins have identified through the interaction detected with a single kindlin; kindlin-2 has been used most frequently in this regard. However, such interactions are likely to extend to at least one and perhaps both other kindlin family members. We discuss several of these interaction as we consider the role of the individual kindlins in breast cancer.

### Associations and functional roles of kindlins in breast cancer

#### Kindlin-1:

K1 is highly expressed in epithelial cells including basal keratinocytes within skin, periodontal tissue and colon being major sites of its expression [37]. Mutations in the K1 gene give rise to a rare autosomal recessive disorder (<200 reported cases), referred to as Kindler Syndrome (KS), which derives its name from the English physician, Theresa Kindlin, who first reported the disorder in 1954 is one of the epidermolysis bullosa diseases and is associated with poikiloderma, skin blistering, photosensitivity and skin atrophy in infants with symptoms changing during patient aging. Deletion of the *FERMT1* gene in mice give rises to many of these same abnormalities including skin atrophy and compromised intestinal barrier function, and results in perinatal lethality [45]. In general deficiencies of kindlin family members in mice closely parallels symptoms observed in human kindlin deficiencies [31].

The rarity of KS precludes drawing interconnections to breast cancer. As of 2012, only two cases of breast cancer in KS patients had been reported [46,47]. However, several studies have identified the presence and role of Kindlin-1 in breast cancer and cancers of other tissues [31,33,34,48]. Azorin et al. [49] detected K1, as well as K2, in human breast tumor cells and suggested that they played distinct and complementary roles within these cells. K1 was involved in supporting cell invasion while K2’s primary influence was on cell spreading. From analysis of human breast cancer tissue, high K1 levels were associated with poorer patient outcomes.

The relationship between K1 and metastasis to lungs was examined by Sin et al. [50], who found a relationship between K1 expression levels in various cancer types metastasizing to lung compared to normal tissue. Kindlin-1 expression was associated with metastasis-free survival for both breast and lung adenocarcinoma. In *in vivo* metastasis, these investigators found that overexpression of K1 was associated with epithelial-mesenchymal transition and transforming TGFβ signaling. K1 reduction in an orthotopic mouse model significantly inhibited breast tumor growth and metastasis to lungs. Additional support for the role of K1 in metastasis was derived by Sarvi et al. [51] in the polyoma virus middle T-driven mouse model of mammary tumorigenesis. Deletion of K1 in the mammary epithelium significantly reduced lung metastasis. This effect of K1 was attributed to its regulation of integrin activation and cell adhesion in the metastatic niche.

Landemaine et al. [52] analyzed the gene expression patterns from 23 breast cancer metastasis from both lung and non-lung tissues and identified a 21-gene signature. Expression patterns of 16 of these were compared to 72 primary breast tumors, and six genes were identified as being predictive of lung metastases. K1 was one of these six genes. However, Culhane and Quackenbush [53] suggested that these analyses may have excluded some confounding factors and may have been influenced by the molecular subtypes of the breast cancer tumors.

#### Kindlin-2:

K2 (*Fermt2*; chromosome 14q22.1) is broadly expressed in most cells of non-hematopoietic origin. K2 loss in mice results in embryonic lethality [54,55], and no K2 deficiency have been reported in humans to date. Like other members of the kindlin family, K2 binds to cytoplasmic tails of β-integrin subunits to promote integrin activation and function [6,56]. However, K2 interacts with numerous diverse binding partners including actin to regulate intracellular cytoskeleton organization [57], clathrin to control endocytosis [58], β-and γ-catenin to regulate Wnt signaling and adherens junction stabilization [59–61], p53 to regulate cell cycle [62] and Epidermal Growth Factor Receptor (EGFR) to support growth factor signaling [63]. Many of these functions assigned to K2, such as regulation of p53, Wnt and EGFR signaling pathways, are associated with cancer progression and metastasis. Indeed, multiple reports demonstrate that K2 is upregulated and promotes growth and metastasis of various cancers in humans and mice including not only breast cancer [62,64,65], but also prostate [66,67], kidney [68], pancreas [69], lung [70], liver [71], brain [72], esophagus [73] and stomach cancer [74].

In breast cancer tumors, K2 expression levels are positively correlated with their aggressiveness. Oncomine analyses of microarray data from 3,455 breast cancer patients revealed that K2 mRNA was increased by 2.537-fold in invasive as compared to noninvasive ductal breast carcinoma, and high K2 expression correlated with a poor relapse-free survival. [63] K2 has been demonstrated to be a biomarker for lymph node metastasis in breast cancer as its expression was enhanced in lymph nodes of patients with metastatic breast cancer [65]. Liu et al. have identified K2 as a risk factor for poor survival in metastatic breast cancer patients [75]. Our own analysis of the Oncomine database showed that K2 was significantly upregulated in breast cancers compared to normal breast tissue and was in the top 3% of upregulated genes in breast cancer [64]. Also, interrogation of the Kaplan-Meyer Plotter breast cancer dataset established that increased K2 expression correlated with reduced survival in breast cancer patients [64]. K2 is associated with many of the oncogenic properties of breast cancer cells including survival, proliferation, adhesion to extracellular matrix, migration and invasion, immunosuppression, resistance to radiation and chemotherapy, and these associations are discussed below.

### K2 promotes EMT

Among a variety of biological factors contributing to breast cancer metastasis, the epithelial-to-mesenchymal transition (EMT) plays a major role. EMT is a process by which epithelial cells lose their polarity and cell-cell junctions, become mesenchymal stem cell-like, and acquire migratory and invasive properties. Under physiological conditions, EMT is crucial in embryo development and regeneration. [76,77] In cancer, EMT liberates carcinoma cells from primary tumors by downregulating E-cadherin and inducing an invasive phenotype. In addition, EMT confers cancer cells with resistance to oncogene-induced senescence and chemotherapy as well as inducing multiple immunosuppression and evasion of apoptosis mechanisms [78–80]. Numerous studies demonstrate that K2 promotes EMT in breast cancer through several mechanisms. K2 has been reported to associate and stabilize DNA methyltransferase I (DNMT1) and enhance its occupancy and methylation of the E-cadherin promoter, ultimately leading to suppression of E-cadherin expression, disruption of adherens junctions and release of cancer cells from solid tumors. Also, K2 expression levels correlate positively with expression of DNMT1 in patients with invasive breast cancer [81].

Wnt/β-catenin signaling pathway plays an important role in tumor progression by regulating EMT and the stem cell-like phenotype of cancer cells [77,82,83]. K2 forms a transcriptional complex with TC4 and β-catenin which enhances Wnt signaling during breast cancer progression. [59] Moreover, Li et al. [60] demonstrated that overexpression of K2 in the mammary glands of mice promoted tumor formation and growth through activation of the Wnt/β-catenin signaling pathway.

MiR 200 family inhibits EMT by targeting ZEB1/ZEB2 transcription factors, and miR 200 loss is crucial to tumor progression [84,85]. Interestingly, K2 promotes EMT and breast cancer progression via epigenetic silencing of the miR 200 gene family. K2 was shown to interact with DNA methyltransferase 3A (DNMT3A) at the miR200b promoter leading to CpG island hypermethylation and inhibition of miR-200b expression [86]. We reported that K2 knockdown in human MDA-MB-231 or mouse 4T1 breast cancer cells inhibits their invasive potential by suppressing invadopodia formation and degradation of extracellular matrix [86]. When injected into the mice, the K2 KO breast cancer cells showed significantly reduced metastases to the lungs in spontaneous and experimental metastasis models. Inhibition of metastasis correlated with suppression of EMT and downregulation of EMT genes: N-cadherin, vimentin and fibronectin in K2 KO tumors. Interestingly, we demonstrated that K2 expression and breast cancer metastasis are downregulated by miR-200b, a master suppressor of EMT, which specifically targets a conserved seed sequence in the 3’UTR of the mouse and human K2 genes [87]. These data together with the findings of Yu et al. [86] indicate that there is K2/miR-200b negative feedback loop that regulates EMT and metastasis: miR-200b inhibits K2 expression suppressing EMT, and K2 downregulates miR200b promoting EMT.

TGF-β suppresses tumor formation and growth, but with tumor progression it switches its behavior from a tumor suppressor to a tumor promotor by augmenting tumor proliferation, migratory/invasive phenotype and metastasis. Indeed, numerous studies have documented this “TGF-β paradox” and have demonstrated that TGF-β is a key regulator of EMT in cancer cells. In breast cancer, K2 is also implicated in TGF-β mediated induction of EMT. We have demonstrated that K2 not only upregulates production and secretion of TGF-β from highly invasive breast cancer cells but also is crucial in TGFβ1-induced signaling as phospho-SMAD3 levels were significantly decreased in the K2-deficient MDA-MB-231 and 4T1 cells [64].

### Role of K2 in senescence and cell cycle regulation

Cellular senescence is key to tumor suppression and is maintained by two well-characterized tumor-suppressor pathways, p53/p21 and p16INK4a/pRB [88]. Many cancer cells have mutations in p53/p21 and/or p16INK4a/pRB, which allows them to escape senescence. Interestingly, Sossey-Alaoui et al. [62] demonstrated that ablation of K2 in human (MDA-MB-231) or mouse (4T1) breast cancer cells induced a senescent phenotype as evidenced by their enhanced polynucleation and increased number of cells arrested in the G1 phase of the cell cycle. Also, K2-deficient breast cancer cells showed enhanced expression of two senescence markers, p21 and SerpinB2 *ex vivo* and in mouse tumor xenografts derived from these cells. p21 and SerpinB2 are p53-responsive genes as p53 interacts with their promoters and increases their expression to induce arrest of cancer growth [89,90]. Mechanistically, we found that K2 directly interacts with p53 and prevents its binding to the promoters of p21 and SerpinB2, thereby promoting breast cancer cell proliferation and tumor growth. Thus, loss of K2 expression bypasses the inhibitory effect of p53 thereby enabling it to bind to the p21 and SerpinB2 promoters and upregulating their expression to induce senescence [62]. This anti-senescence action of K2 does not require K2/integrin interactions as mutation of its integrin binding site did not compromise this function.

In addition to suppression of p53, K2 maintains MT (microtubule) spindle integrity during mitosis. K2 promotes mitotic spindle assembly by maintaining α-tubulin acetylation in mitotic spindles. It does so by inhibiting the MT-associated deacetylase histone deacetylase 6 (HDAC6) downstream of integrin-dependent activation of AKT Ser/Thr kinase (AKT), glycogen synthase kinase 3β (GSK3β) and paxillin signaling pathways. Hypoxia increases levels of miR-138 in MDA-MB-231 breast cancer cells, and downregulation of K2 leads to profound spindle abnormalities and delayed mitosis [91]. However, Zhao et al. reported that mitotic MCF-7 breast cancer cells overexpressing K2 had multiple chromosomal defects compared to control cells leading to significant changes in gene copy numbers and genome instability [92]. Nevertheless, overexpression of K2 in MCF-7 cells increased their proliferation and the number of cells in the G2/M phase as well as protecting them from apoptosis while K2 knockdown reversed these effects. *In vivo*, overexpression of K2 in MCF-7 cells promoted tumor formation in implanted xenografts while K2 downregulation reduced tumor growth in mice [92].

### K2 modifies the tumor microenvironment

Tumor progression is strongly determined by complex interplay between tumor cells and host cells in the tumor microenvironment. Immune cells, including macrophages, are important components of the tumor microenvironment. In breast tumors, macrophages are “educated” by tumor cells to adopt a pro-tumorigenic phenotype. Such macrophages contribute not only to tumor growth and metastasis but also to host immunosuppression [93–95].

K2 supports tumor growth by modulating the interaction between tumor and tumor microenvironment. Our group described K2-dependent paracrine TGF-β/CSF-1/EFG signaling pathway that is crucial for growth and progression of breast cancer tumors [64]. K2 enhances TGF-β-induced production and secretion of Colony Stimulating Factor-1 (CSF-1), which stimulates macrophage chemotaxis into the tumor microenvironment as well as their polarization to the M2 immunosuppressive phenotype. Macrophages promote breast cancer tumor growth by secreting various growth factors including EGF, which is recognized by tumor EGF receptors to stimulate tumor cell proliferation and migration [64]. However, this step is also dependent upon K2 since interaction of EGF with EGFR is required for receptor stabilization and EGFR-induced intracellular signaling [63].

Recent studies have implicated K2 in regulation of extracellular matrix stiffness, including in the tumor microenvironment. Xue et al. [65,96] demonstrated that K2 together with hypoxia-inducible factor-1 (HIF-1α) increased breast cancer matrix stiffness, and the two are highly expressed in invasive breast cancer and correlate with tumor stiffness. K2 enhanced matrix stiffness by upregulating collagen production, which is achieved by increasing expression of collagen prolyl 4-hydrolases via an integrin/FAK pathway. The relationship between K2 and matrix stiffness in multi-dimensional. Guo and colleagues [70] showed that extracellular matrix stiffening stimulated K2 translocation to mitochondria and its interaction with pyrroline-5-carboxylate reductase 1 (PYCR1) leads to enhanced proline synthesis supporting cancer cell proliferation. Depletion of K2 reduced PYCR1 and proline levels and reduced fibrosis *in vivo* leading to substantial inhibition of tumor growth.

#### Kindlin-3

K3 is highly expressed in the hematopoietic system; however, there are surprisingly only limited reports concerning its involvement in blood cell cancers. K3 was found to be associated with the pathology of chronic myeloid leukemia [97,98] and acute myeloid leukemia[99,100]. Qu and colleagues [97] showed that K3 regulated the proliferation of human chronic myeloid leukemic K562 cells through regulation of c-Myc protein expression and controlled tumor growth of these cells in a xenograft model. Using a murine model of chronic myeloid leukemia, Krenn et al. [98] demonstrated that loss of K3 expression in leukemic stem cells in bone marrow led to release of these cells into the circulation and impaired their proliferation and survival in secondary organs, which suppressed chronic leukemia progression and metastasis. In another study, Wu and colleagues reported that K3 increased in patients after complete remission of acute myeloid leukemia [99]. Subsequently, Qin and colleagues reported that miR-4792 directly downregulated K3 expression in acute myeloid leukemia cells [100], and this downregulation inhibited cell proliferation and invasion and induced apoptosis of the myeloid leukemia cells.

Several studies have identified K3 in solid tumors but its role as a tumor promoter or tumor suppressor remains controversial. In melanoma cells, tumor suppressor functions were assigned to K3 as its downregulation in human melanoma cell lines SKMEL28 and M10 enhanced their metastatic potential [101]. In a separate study, the Mourah group showed a role of K3 in regulating β1 integrin function in M10 human melanoma cell line [102]. Overexpression of K3 in melanoma cells also leads to abnormal Rac1 and RhoA activation, which impedes cell migration [103].

Our published studies have identified K3 and a single phosphorylation within it as a promoter of breast cancer progression and metastasis [35,104]. The relationship between K3 and breast cancer in mouse models and human tissues was examined [104]. Compatible with its role as a tumor promoter in breast cancer, K3 was identified as one of the top 3% of gene products elevated in breast cancer (p< 10^−12^) in several Oncomine (www.oncomine. com) datasets [104]. *In vitro* analyses determined that K3 stimulates breast cancer cell migration and invasion. Mechanistically, K3 overexpressing cells showed a marked increase in VEGF production, which was attributed to enhanced expression of the transcription factor Twist and enhanced β_1_ integrin activation.[104] *In vivo*, K3 overexpression in a breast cancer cell line increased primary tumor growth and lung metastasis, a consequence of enhanced tumor angiogenesis consistent with increased VEGF production and macrophage recruitment, downstream of Twist, which also activates the EMT program [104].

In a prior study, Bialkowska et al.[105] had identified agonist-induced K3 phosphorylation on T^482^ and/or S^484^ in hematopoietic cells and had established a crucial role of K3 phosphorylation in integrin-induced signaling. In a more recent work, K3 phosphorylation was found to exert a tumor promotor activity in breast cancer cell lines [35]. The K3-induced *in vitro* and *in vivo* effects on breast cancer progression were blunted by K3 bearing T^482^S^484^/AA mutations, including reduced K3-induced tumor angiogenesis and macrophage recruitment *in vivo*. Twist expression was also significantly blunted *in vitro.* Tumors derived T^482^S^484^/AA K3-expressing cells were markedly smaller than those derived from cells expressing wild-type K3 [35]. It is worthwhile noting that another group has identified K3 phosphorylation as an important regulator of its function in leukocyte trafficking [106], and we used a phospho-specific antibody to implicated K3 phosphorylation site within breast cancer cells [35].

In contrast, other groups investigated the role of K3 in breast cancer and have concluded that it exerts tumor suppressor activity [101]. While Djaafri and colleaguescould readily detect K3 in MDA-MB-231 cells, it was present at lower levels than in normal breast epithelial cells and proposed that K3 was downregulated in several solid tumors by a mechanism involving gene hypermethylation and deletions [101]. In *in vivo* experiments, they demonstrated that K3 knockdown in breast cancer cells markedly increased metastasis, in agreement with the *in vitro* increase in malignant properties associated with K3 knockdown. The metastatic phenotype of K3 knockdown cells involved changes in β3-integrin activation including decreased phosphorylation of β3-integrin cytoplasmic tail and suppressed integrin-talin interaction accounting for a more migratory and invasive phenotype. It is worth noting that the authors did not report the effects of K3 overexpression on tumor progression and metastasis in mouse models, so the effect of elevated K3 was not established [101]. The basis for the different findings between our group and those of Djaafro et al. [101] are not at all clear. It is more fundamental than a difference in the specificity of the kindlin-3 antibodies used since both groups supported their findings at the mRNA levels.

Yet another group [49] investigated expression profiles and functions of kindlin family in breast cancer, and they were unable to detect K3 in several breast cancer cell lines including MDA-MB-231 cells [49], which is in disagreement with previous findings [35,101,104]. In a patient-derived xenograph mouse model, K3 was exclusively detected in mouse cells, suggesting an expression in the tumor microenvironment [49]. In patient tissues, the highest K3 expression levels were observed in triple negative and ERBB2 breast cancer tumors where it correlated with amount of tumor infiltrating leukocytes and poor patient prognosis [49].

## Kindlins as Therapeutic Targets

With regard to K1 and K3, current data is too limited or too controversial to consider them as therapeutic targets for breast cancer. A vast number of reports have established K2 is heavily involved in breast cancer growth, progression and metastatic dissemination. Indeed, K2 has been associated with almost every event in breast cancer progression as it promotes cancer cell proliferation, survival, adhesion, migration, invasion and EMT, thereby establishing a pro-tumorigenic microenvironment. Thus, it is tempting to consider interference with K2 as a therapeutic target for many cancers including breast cancer. This consideration must be tempered by the knowledge that K2 is indispensable in most normal non-hematopoietic cells in the body. However, a 50% reduction of K2 in K2+/− mice does not lead to an overt spontaneous phenotype, and partial reduction of K2 in breast cancer cells with shRNA interference does blunt tumor growth. Thus, targeted and safe delivery of K2 inhibitors to tumor cells could and should be explored as a potential breast cancer therapy.

## Figures and Tables

**Figure 2.**
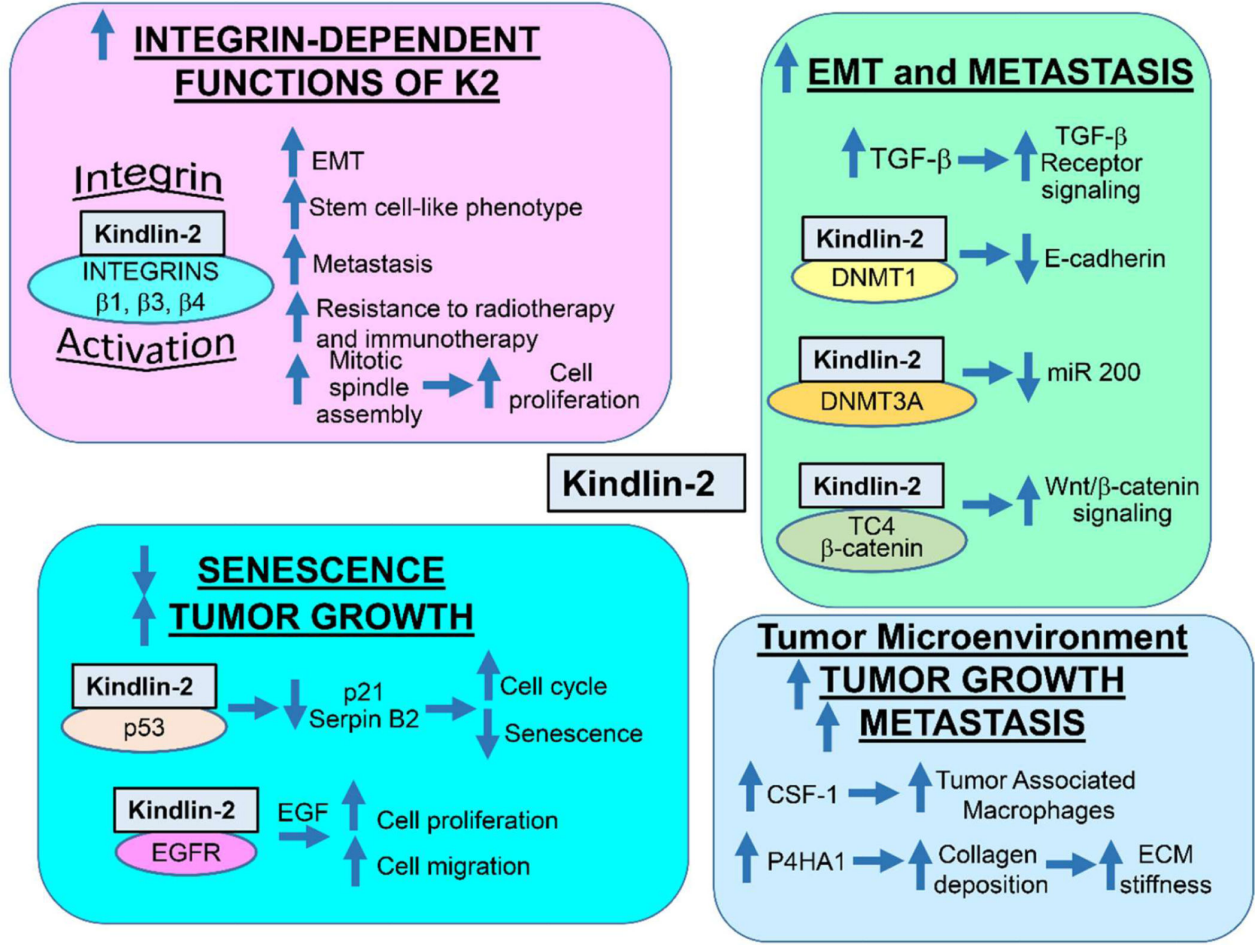
A highlighted summary of the identified functions of Kindlin-2 in the development, progression and metastasis of breast cancer.

## Abbreviations:

K1: Kindlin-1
K2: Kindlin-2
K3: Kindlin-3
KS: Kindler Syndrome
FERM: 4.1-ezrin-radixin-moiesin
TGFβ: Transforming Growth Factor β
